# Editorials

**Published:** 1894-03

**Authors:** 


### HOMŒOPATHIC PHYSICIAN,

A MONTHLY JOURNAL OF

HOMŒOPATHIC MATERIA MEDICA AND CLINICAL MEDICINE.

"If our school ever gives up the strict inductive method of Hahnemann, we are lost, and deserve only to be mentioned as a caricature in the history of medicine."—CONSTANTINE HERING.

Vol. XIV. MARCH, 1894. No. 3.

### EDITORIALS.

A WORD WITH OUR SUBSCRIBERS.—It has been widely circulated and is generally understood that negotiations are now in progress for a consolidation of THE HOMŒOPATHIC PHYSICIAN with *The Medical Advance*.

As a consequence I have received a number of inquiries from interested friends desiring definite information if such a union were probable. I desire to reply that, though such a union might be advantageous in some ways, in others it is deemed unadvisable.

THE HOMŒOPATHIC PHYSICIAN will consequently continue to be published as heretofore, by me, along the same lines that have brought about its present prosperity.

WALTER M. JAMES, M. D.,
*Editor and Publisher.*

---

THE WARNING OF CONSTANTINE HERING.—Constantine Hering was both a savant and a philosopher; as a savant he knew the value of laws and of facts based thereon; as a philosopher, he had studied the history of man's past life, during the centuries gone by, and had learnt that all theories perish, and that only facts based upon Nature's immutable laws remain.

In consequence of this knowledge, Dr. Hering once proclaimed that "If our school ever gives up the strict inductive method of Hahnemann, we are lost, and deserve only to be mentioned as a caricature in the history of medicine."

What is this "strict inductive method of Hahnemann" that Dr. Hering considered absolutely necessary to the life of Homœopathy? In plain every-day terms, it is simply the proving of drugs upon the healthy and prescribing these proven drugs for similar symptoms in the sick; this is the system of medicine founded by Hahnemann and one that he declared, judging from his own knowledge and experience, could be so perfected that one day the curing of the sick would become as certain as it is that two and two make four! Is medicine now in that condition of certainty? If not, where is the fault? Was Hahnemann wrong in his belief? Have his successors, in medicine, followed out his "strict inductive method" and found it could not be so perfected? If so, then we must acknowledge that Hahnemann was wrong. On the other hand, if his successors have not seriously tried to perfect medicine along the lines he pointed out, and they are the only ones that any man has yet ventured to prophesy such a glorious success upon—then his followers have proven themselves unworthy of the legacy bequeathed them. Not only so, but they have proven themselves false to humanity! If the laity, at large, knew there existed a large body of men, who possessed a method of curing humanity, its many ailments, and also of preventing many others from ever developing, and yet neglected its study and development, the mass of mankind would arise in its just indignation and hiss these recreant doctors from the face of the earth!

This is very strong language, but it seems none too strong for the wrong that is being perpetrated. No one who has seen a dear friend or relative sink into the grave because "everything that science could suggest" could not rescue it, would consider any language too strong in which to characterize physicians who are thus trifling with humanity.

But, perhaps, many will sneer at these words, and declare that "science" is progressing and medicine with it. Is this true? What is "science" teaching the old school doctors or their petty imitators in the new school? Are the deaths lists decreasing? Is any one counting the numbers of deaths from the "grip;" or, even worse, its many victims who take their own lives in sheer despair?

Not only are the deaths from this grip very few amongst the patients of true Hahnemannians; but one could safely declare that not one suicide has been found from amongst their patients. Is not this something? Perhaps, again, some one will declare that the system of medicine has nothing to do with suicide? Perhaps not. The writer once had a lady who was suffering greatly from the results of allopathic over-dosing; on his first visit, for mental symptoms, he prescribed $Aurum^{200}$; some days later, the lady told him that she had had in her possession, at the time of his first visit, a bottle of laudanum, which she had purchased for the purpose of poisoning herself; her sufferings, for many, many years had been too much for her and she had made up her mind to release herself from their tortures. But after taking the homœopathic medicine she had felt better, and had thrown away the laudanum. She did not consider that those little pellets had saved her from a great crime. Do you, reader?

But this digression upon the grip has taken us away from our original subject; which is this warning of Dr. Hering. Our case stands thus: Hahnemann, a man so learned and so good that allopathic bigotry could suggest nothing against him either as a physician or as a man. It could only sneer and declare him "a visionary." This man discovered a law governing the action of drugs; and he thoroughly tested it. Finally so convinced was he of its truth and its power, in consequence of its truth, that he declared it would, when perfected, enable physicians to cure the sick with mathematical certainty. Numbers of men, learned and unlearned, have tried this law of healing, in the manner advised by Hahnemann, and, almost to a man, have agreed with him. Only those who have not tried this method, in the proper manner, declare it to be unsuccessful. Now as testimony is usually considered in all matters, which of these parties shall be believed? No sensible man can doubt. If then, Hahnemann was correct, why is the homœopathic school of to-day, so entirely neglecting to study and to develop his method?

It may be that some one will say that Hahnemann's strict inductive method is not being neglected! Does any one make this declaration? If so, we ask, has he read any recent "work on Homœopathy"? But we beg pardon; the recent "works" are never credited to "Homœopathy" and it is well, for there is none of it in them. Has our supposed objector seen any of the current literature which passes through the post-office as homœopathic medical journals? If any one has carefully looked over any of the recent literature that circulates throughout the country, can he truthfully deny that the so-called homœopathic school has not already become "a caricature in the history of medicine"?

The remedy? If it be acknowledged that this picture of the utter degeneracy of Homœopathy be not overdrawn, it may not be out of place to ask for the *remedy*. Many might be suggested, but one will suffice for the present. As we look around us we see the medical schools, the text-books, and the so-called homœopathic journals teaching a debased, inefficient system of practice; it is therefore our duty (and by "our" each and every individual physician is meant) to exert ourselves in every way possible to counteract these pernicious influences. It is not enough to practice pure Homœopathy; one should do more—try to teach others to do the same. This can be done, in the largest measure, by strong support of true homœopathic journals—support both by pen and purse. Each true practitioner should consider it his duty and his privilege to furnish several able articles every year to the journal he considers the ablest exponent of Hahnemannian Homœopathy. And as for the thriving practitioner who is too selfish, too mean, or too indifferent to pay his subscriptions, he is too despicable and too far sunken in the scale of humanity to be aroused by any *words;* what might be accomplished by *blows* is even doubtful.

Is it not time to cease our petty, silly quarrels? Are we not in the position of seamen on a sinking vessel; and what would be thought of seamen who indulged in childish quarrels rather than man the pumps or stop the leaks? Humanity would declare they fully merited their watery grave! And so will the historian of the future say of homœopathic physicians. He will declare, as he studies the writings of Hahnemann and his great followers, that they left a legacy too grand and too rich for the feeble hands that came after to support and develop; and they will become "a caricature in the history of medicine."

E. J. LEE.

---

THE NEW YORK HOMŒOPATHIC UNION, through its Secretary, Dr. Wilcox, has sent for publication in THE HOMŒOPATHIC PHYSICIAN a full stenographic report of its proceedings at the meeting of December last. This report is exceedingly interesting, and it would be a good thing to publish it in full. Unfortunately it is too bulky for the space at disposal. It has therefore been much abridged. The portion given is composed only of extracts of the most interesting passages of the discussion.

